# Severe Myocardial Infarction Caused by Excessive Cannabis Consumption

**DOI:** 10.7759/cureus.14643

**Published:** 2021-04-23

**Authors:** Ramia Bougrine, Mohamed Boutaybi, Soumia Boulouiz, Noha Elouafi, Zakaria Bazid

**Affiliations:** 1 Cardiology, Mohammed I University/Mohammed VI University Hospital, Oujda, MAR; 2 Cardiology, Mohammed I University/Mohammed VI University Hospital/Epidemiological Laboratory of Clinical Research and Public Health, Oujda, MAR

**Keywords:** cannabis use, inferior-posterior myocardial infarction, coronary artery disease, coronary artery vasospasm

## Abstract

The cardiovascular effects of cannabis are not well known. Cannabis use has been shown to cause arrhythmia, including ventricular tachycardia, sudden death, and increase in the risk of myocardial infarction (MI). We report a 62-year-old woman with chronic cannabis abuse inducing MI complicated by cardiogenic shock, chronic dilated cardiomyopathy, and atrial fibrillation.

## Introduction

Cannabis is one of the most popular illicit drugs in the world. It is often consumed for its euphoric and hallucinogenic effects [1], which could be a significant contributing factor to a cardiovascular event. However, cannabis as a cardiovascular factor should not be overlooked. Even though the exact role of this drug in the pathogenesis of coronary heart disease remains poorly understood, the legalization of cannabis would be a good demographic/sociological issue to discuss, which could increase the cardiovascular events in our society.

We report a case of severe myocardial infarction (MI) in an elderly woman chronically addicted to cannabis.

## Case presentation

A 62-year-old woman with a history of tobacco abuse (30 pack-years) and chronic cannabis use, presented with severe retrosternal chest pain with peripheral vascular collapse (weak peripheral pulses, pale skin), sweating, and vomiting after 30 min of excessive cannabis consumption, i.e. more than 15 cigarettes.

**Figure 1:**
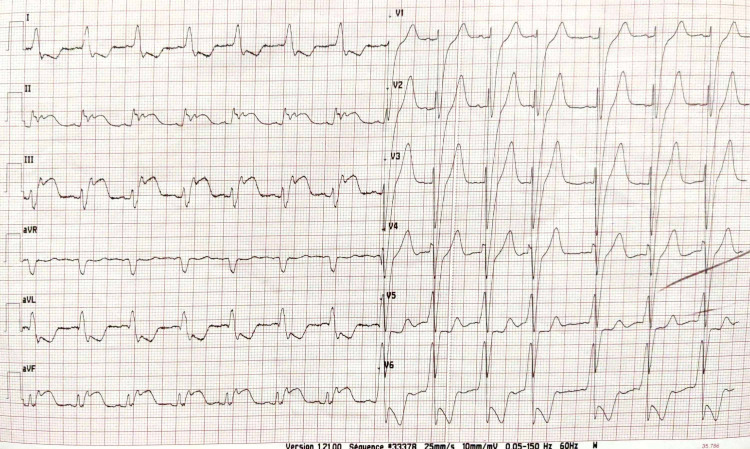
Electrocardiogram with ST-segment elevation in inferior leads.

**Figure 2:**
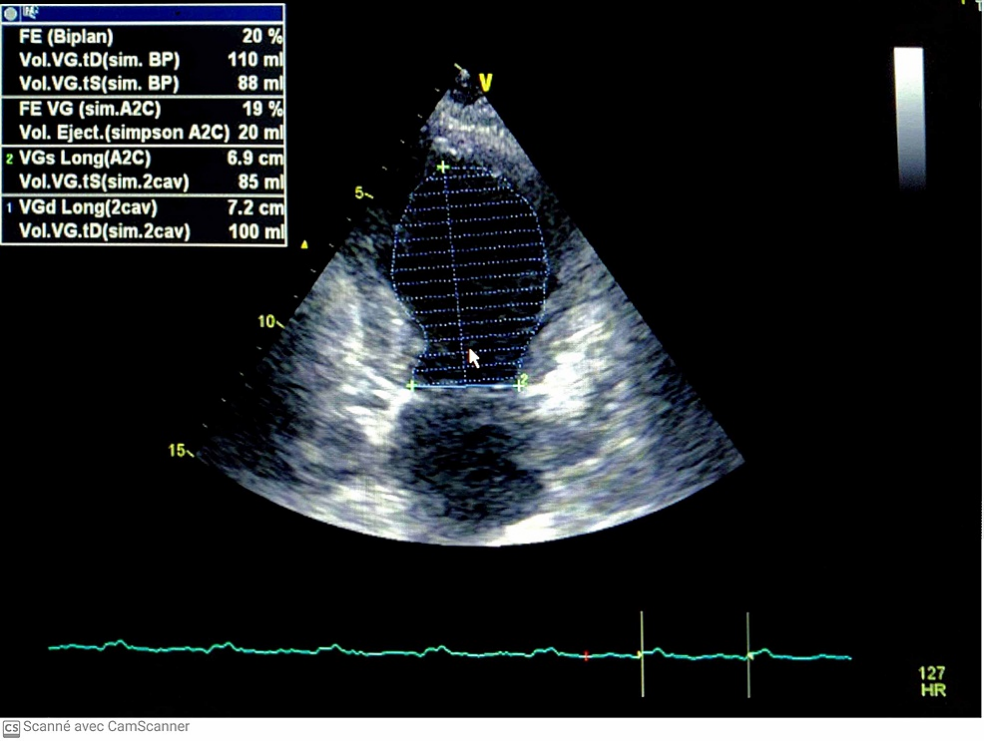
The echocardiography showed a dilated cardiomyopathy with severe EF 20%. EF, ejection fraction

**Figure 3:**
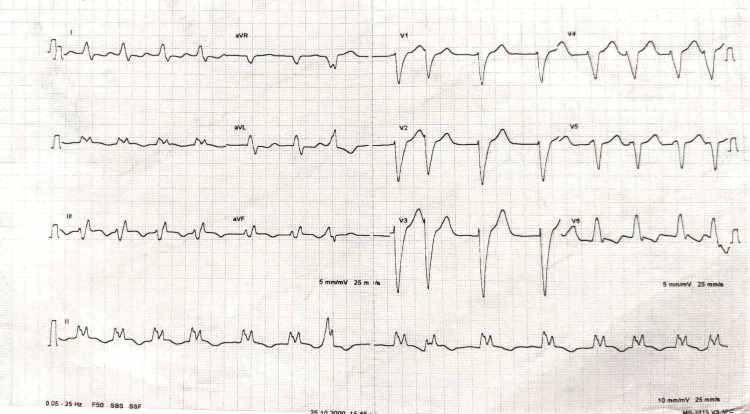
Regression of ST-segment elevation in inferior leads with atrial fibrillation.

**Figure 4:**
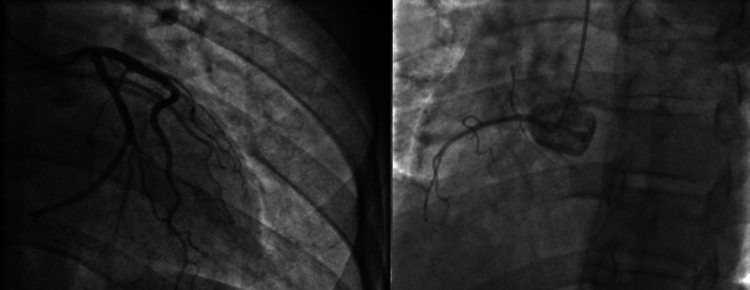
A coronary angiogram showed normal coronary arteries without stenosis (left dominance).

The physical examination found a pale, agitated patient, with a blood pressure limit of 100/60 mmHg; the cardiovascular examination was normal; electrocardiogram (ECG) revealed ST-segment elevation in inferior and basal leads (Figure 1). An echocardiogram was performed, revealing dilated cardiomyopathy with global hypokinesia, a severely reduced ejection fraction (EF) of 20%, and associated right ventricular dysfunction (Figure 2). After a drop in blood pressure, the patient was started on inotropic agents (dobutamine 10 gamma/kg/min). We noted the disappearance of chest pain and regression of ST-segment elevation after hemodynamic state stabilization (Figure 3). A coronary angiogram which was performed at hour eight of chest pain showed normal coronary arteries without stenosis (Figure 4). The ECG monitoring showed atrial fibrillation at 100 bpm. Laboratory measurements showed elevation of cardiac biomarkers troponin (41600 ng/mL, the upper limit of normal < 26 ng/mL).

The patient was admitted to the ICU, and started on an IV vasodilator and inotrope agent. The hemodynamic status was stabilized after 48 h, with the persistence of severe ventricular dysfunction and atrial fibrillation. The patient was discharged after a week with diltiazem, ACE inhibitor, diuretic; anticoagulation by rivaroxaban 20 mg was initiated. 

Clinical follow-up after three and six months later was favorable with nonrecuperation of a normal ventricular function.

## Discussion

Cannabis is a common drug derived from the Cannabis sativa plant. Its physiological effects are mediated by the interaction of THC D9-tetrahydrocannabinol with the endocannabinoid system with at least two cannabinoid membrane receptors (CBR1 and CBR2). CBR1 is the most expressed in the central nervous system (brain, heart, blood, spleen, etc.), peripheral (artery, immune system), and autonomic nervous system, which acts by activation of the sympathetic nervous system, as well as by inhibition of the parasympathetic nervous system [2-3].

The relationship between cardiovascular events remains unknown. Regular cannabis consumption may increase the level of catecholamines, the level of carboxyhemoglobin, which limits the capacity of oxygen transport. A mismatch between increased oxygen demand and decreased myocardial oxygen supply can promote the onset of coronary syndrome, orthostatic hypotension, increased blood pressure, and cardiac workload [4]. Cannabis has also been described as having procoagulant effects since both receptors are expressed on the cells of the platelet membrane [5]; the other effect was tachyarrhythmia, supraventricular or ventricular can be explained by the high level of adrenaline following cannabis abuse [6]. Cannabinoids have also been shown to reduce myocardial contractility through effects mediated by CBR1 [7].

Myocardial infarction was also reported. Thrombus formation, coronary vasospasm, and coronary artery dissection are the three proposed mechanisms described in literature [8-9]. Casier et al. [3] reported that cannabis abuse was associated with severe coronary syndrome due to coronary spasm and MI with a total artery occlusion. Deharo et al. [10], Ghannem et al. [11], and Yurtdaş et al. [12] reported cases of excessive cannabis use induced MI after an exercise in healthy young men with a thrombotic occlusion. Finally it is still obscure, if cannabis contributes to coronary diseases in the same way as smoking [13], but these case studies indicate that cannabis can be a risk factor for MI in addicted persons.

In our case, the angiography revealed normal coronary arteries. The diagnosis of coronary vasospasm was accepted given the context and other cannabis complications. Also, due to the advanced age of our patient, it would be possible to have an associated atherosclerosis coronary lesion.

This case highlights the potential danger of cannabis especially in patients with chronic cannabis consumption [14].

The prevalence of coronary diseases caused by the consumption of cannabis may increase due to the legalization of this drug, which will increase potentially the incidence of MI in consumer patients [15].

## Conclusions

This case highlights an unusual case in elderly women with excessive use of cannabis causing severe cardiovascular complications: MI with shock, arrhythmia, which should be systematically investigated in patients’ consumers with a coronary syndrome, especially without a cardiovascular risk factor. Coronary vasospasm and intracoronary thrombus appear to be the two main mechanisms involved. 

The treatment is essentially preventative and relies on stopping cannabis use, but with the legalization of cannabis consumption, we should be close to receiving more coronary syndrome and its complications.
